# Off-Label Use of Sodium Valproate for Schizophrenia

**DOI:** 10.1371/journal.pone.0092573

**Published:** 2014-03-24

**Authors:** Einav Horowitz, Lisa Carroll Bergman, Charna Ashkenazy, Irit Moscona-Hurvitz, Haya Grinvald-Fogel, Racheli Magnezi

**Affiliations:** 1 Israeli Center for Technology Assessment in Health Care, Gertner Institute, Haim Sheba Medical Center, Ramat Gan, Israel; 2 Public Health and Health Systems Management Program, Bar Ilan University, Ramat Gan, Israel; 3 Yehuda Abarbanel Mental Health Center, Bat Yam, Israel; 4 Teva Pharmaceutical Industries, Ltd., Petach Tikva, Israel; The Nathan Kline Institute, United States of America

## Abstract

**Background:**

Off-label use of a drug not according to its regulatory labeling has become common in medicine, especially in the field of psychiatry. Mood stabilizers are intended to be used to attenuate mood fluctuations in bipolar disorder, but their use has spread to patients with schizophrenia, as it provides greater control of impulsivity and aggressiveness. Sodium valproate is one of the most frequently used mood stabilizers in psychiatry. This study determined the prevalence of off-label use of sodium valproate for schizophrenia and schizoaffective disorder in Abarbanel Psychiatric Hospital and the demographic and clinical characteristics associated with its use.

**Methods:**

Retrospective study of patients hospitalized in 2011–2012 with a diagnosis of schizophrenia or schizoaffective disorder in one of three general psychiatric wards.

**Results:**

Valproate use was significantly lower in the geriatric group (11.6% vs. 20.1%, chi square  = 4.7, p = .03), in patients with schizophrenia (14.1% vs. schizoaffective disorder (35.2%), chi square  = 29, p<.001) and in patients receiving both atypical and typical antipsychotics (23.3% vs. 16.4%, p = .04). In multivariate logistic regression analysis, diagnosis and the *combination* of atypical and typical antipsychotics predicted the use of sodium valproate. The number of other medications prescribed did not predict sodium valproate use.

**Conclusions:**

Off-label use of sodium valproate in psychiatric patients with schizophrenia or schizoaffective disorder is extensive, especially in younger patients and those with schizoaffective disorder. More research is needed to determine whether it is being prescribed appropriately.

## Introduction

Off-label use (OLU) – the use of a drug or a device for a purpose or in a manner outside its regulatory labeling – has become a common practice in many fields of medicine [1]. Radley et al. found that 21% of prescriptions given to patients in general office practices were for off-label indications [2]. Senior clinicians and regulators from a number of European countries and North America regard off-label prescribing as a necessary part of the art of medicine [3].

OLU is particularly common in psychiatry. The lack of clarity regarding the physiological basis for psychiatric disorders, the relative ineffectiveness (and sometimes troublesome side-effects) of various drugs and the symptoms shared by many of the disorders, have led physicians to use drugs approved for one disorder for one for which it is not approved [4]. Leslie et al. reported that more than half of psychiatrists surveyed routinely prescribed psychotropic medications off-label [5]. The phenomenon of off-label prescribing is so common that in some instances, psychiatrists are not aware that they are prescribing off-label [6].

Antipsychotics (typical and atypical) are still the cornerstone of schizophrenia treatment. Many individuals with schizophrenia require a combination of drugs, including different classes of drugs, such as antidepressants, anxiolytics, mood stabilizers and various other medications to treat the side-effects of the neuroleptic treatments. Polypharmacy is common. One-fifth to one-third of individuals with schizophrenia does not respond to antipsychotics [7].

Mood stabilizers are thus named because they attenuate the mood fluctuations of patients with bipolar disorder, but their usage has spread to other psychiatric conditions. Unlike the combination of atypical antipsychotics and mood stabilizers in bipolar disorder, the use of sodium valproate in patients with schizophrenia has limited empirical evidence [8], [9]. It therefore remains an off-label indication. Despite that, the use of mood stabilizers as augmentation therapy for schizophrenic patients has become widespread. The utilization rates of mood stabilizers in patients with schizophrenia range from 15% to almost 50% [1], [10]–[14].

Sodium valproate was originally developed as an anticonvulsant and was later approved for manic episodes associated with bipolar disorder [15], [16]. In the past decade, it became the most frequently prescribed mood stabilizer for patients with schizophrenia, with lithium lagging far behind [1], [10], [11], [14].

The goal of this study was to determine the prevalence of off-label sodium valproate prescription for schizophrenia and schizoaffective disorder in a major psychiatric hospital in Israel and to determine the demographic and clinical characteristics associated with its use.

## Methods

This retrospective study was conducted at the Abarbanel Mental Health Center, the largest psychiatric facility in Israel, using a computerized database. This 300-bed academic hospital covers a population of approximately one million. It provides treatment for individuals with personality disorders, as well as neurotic, psychotic and organic conditions. In addition, the center offers out-patient services, psychiatric rehabilitation and long-term psychiatric day-care. Patients range in age from youth to the elderly.

Patients with a diagnosis of schizophrenia (ICD codes F.20–F.20.9) or schizoaffective disorder (ICD code F.25) hospitalized from 2011 through 2012 in a general ward for men or women or in a geriatric ward were included in this study. If a patient had both diagnoses, the most recent one was used. Patients were excluded if they had a concomitant diagnosis of epilepsy or bipolar disorder, both labeled indications for sodium valproate.

Data extracted from the electronic medical record system included age, gender, hospital admission and discharge dates, diagnosis and treatment. If a patient was hospitalized more than once within the study period, data from the most recent hospitalization were used. Medications used by the patients were grouped into the following categories: typical (first generation) antipsychotics, atypical (second generation) antipsychotics, antidepressants, anxiolytics, mood stabilizers (other than sodium valproate) and sodium valproate.

### Ethics statement

The study was approved by the Abarbanel Hospital Ethics Review Board. The Ethics Review Board granted approval to review the patient records and extract the necessary data. The Ethics Review Board waived the need to obtain individual informed consent because the data were analyzed anonymously.

### Measures

The number of drug types prescribed for a patient (other than sodium valproate) was calculated as a count of the five categories included in the study (typical antipsychotics, atypical antipsychotics, antidepressants, anxiolytics and mood stabilizers), yielding a scale ranging from 0 to 5. Antipsychotic polypharmacy (APP) was defined as a combination of typical with atypical antipsychotics. For logistic regression, dichotomous variables were used to analyze the effect of gender, ward and diagnosis.

### Statistical Analysis

All statistical analyses were performed using the SPSS statistical software, version 20.0. The χ^2^ test for independence was carried out to examine the dependency between the diagnosis and the ward to the use of sodium valproate.

Logistic regression was used to examine gender, diagnosis, ward and number of drug types and APP as potential predictors of the use of sodium valproate. Odds ratios with 95% confidence intervals were calculated for each independent variable.

## Results

Among approximately 1,300 hospitalizations, 604 individuals matched the inclusion and exclusion criteria, 253 in the men's ward, 230 in the women's ward and 121 in the geriatric ward. The average age of the patients was 45.9 (SD 14.7) years. The age range in the men's ward was 18 to 65 years, in the women's ward, 19 to 64 years and in the geriatric ward, from 60 to 85 years. Patient characteristics are presented in Table 1. A total of 482 (79.8%) were diagnosed with schizophrenia.

**Table 1:** Patient demographics and medications by ward.

Characteristics	Ward			Total N = 604
	Men N = 253	Women N = 230	Geriatric N = 121	
**Mean age [SD], (range), years**	38.8 [10.3] (18–65)	42.5 [11.0] (19–64)	67.3 [6.2] (60–85)	45.9 [14.7]
**Gender, % (n)**				
Male	100.0% (253)	0.0% (0)	49.6% (60)	51.8% (313)
Female	0.0% (0)	100.0% (230)	50.4% (61)	48.2% (291)
**Diagnosis, % (n)**				
**Schizophrenia**	86.6% (219)	72.2% (176)	80.2% (87)	79.8% (482)
1. Schizophrenia	68.0% (172)	53.0% (122)	66.1% (80)	61.9% (374)
2. Paranoid	3.6% (9)	0.0% (0)	0.0% (0)	1.5% (9)
3. Hebephrenic	4.0% (10)	10.4% (24)	3.3% (4)	6.3% (38)
4. Catatonic	0.4% (1)	0.9% (2)	0.0% (0)	0.5% (3)
5. Undifferentiated	1.6% (4)	0.4% (1)	0.8% (1)	1.0% (6)
6. Residual	4.7% (12)	0.9% (2)	8.3% (10)	4.0% (24)
7. Simple	0.4% (1)	0.0% (0)	0.0% (0)	0.2% (1)
8. Unspecified	4.0% (10)	6.5% (15)	1.7% (2)	4.5% (27)
**Schizoaffective disorder**	13.4% (34)	27.8% (64)	19.8% (24)	20.2% (122)
1. Schizoaffective disorders	12.3% (31)	21.7% (50)	17.4% (21)	16.9% (102)
2. Depressive type	0.0% (0)	3.9% (9)	0.0% (0)	1.5% (9)
3. Mixed type	0.4% (1)	1.7% (4)	0.8% (1)	1.0% (6)
4. Unspecified	0.8% (2)	0.4% (1)	1.7% (2)	0.8% (5)
**Drug/Drug types** a **, % (n)**				
**Sodium valproate**	20.9% (53)	19.1% (44)	11.6% (14)	18.4% (111)
**Typical antipsychotics** b	61.2% (155)	77.8% (179)	27.3% (33)	60.8% (367)
** Number**				
1	55.7% (141)	64.8% (149)	24.8% (30)	53.0% (320)
2	5.1% (13)	12.6% (29)	2.5% (3)	7.5% (45)
3	0.4% (1)	0.4% (1)	0.0% (0)	0.3% (2)
**Atypical antipsychotics** c	55.7% (141)	65.6% (151)	85.9% (104)	65.6% (396)
** Number**				
1	47.0% (119)	54.3% (125)	76.0% (92)	55.6% (336)
2	8.7% (22)	11.3% (26)	9.1% (11)	9.8% (59)
3	0.0% (0)	0.0% (0)	0.8% (1)	0.2% (1)
**Antidepressants** d	9.5% (24)	3.9% (9)	0.8% (1)	5.6% (34)
** Number**				
1	9.1% (23)	3.5% (8)	0.8% (1)	5.3% (32)
2	0.4% (1)	0.4% (1)	0.0% (0)	0.3% (2)
**Anxiolytics** e	58.9% (149)	42.1% (97)	23.4% (27)	45.2% (273)
** Number**				
1	57.3% (145)	40.4% (93)	20.7% (25)	43.5% (263)
2	1.6% (4)	1.7% (4)	1.7% (2)	1.7% (10)
**Mood stabilizers** f	8.7% (22)	8.3% (79)	9.9% (12)	8.3% (53)
** Number**				
1	8.3% (21)	7.4% (17)	9.9% (12)	8.3% (50)
2	0.4% (1)	0.9% (2)	0.0% (0)	0.5% (3)
**Sleeping pills** g	66.8% (169)	34.8% (80)	79.3% (96)	57.1% (345)

a Totals might exceed 100% because some patients received more than one drug type.

b chlorpromazine, flupentixol, fluphenazine, haloperidol, levomepromazine, penfluridol, perhphenazine, zuclopenthixol.

c amisulpride, aripriprazole, asenapine, clotiapine, clozapine, olanzapine, paliperidone, palperidone, quetiepine, risperidone, sertindole, ziprasidone.

d escitalopram, paroxetine, reboxetine, trazadone.

e buspirone, clonazepam, diazepam, lorazepam, oxazepam.

f carbamazepine, lamotrigine, lithium.

g brotizolam, nitrazepam, zopiclone.

The most commonly prescribed medications were antipsychotics; over 97% of patients received at least one. Geriatric patients tended to receive atypical rather than typical antipsychotics (85.9% vs. 27.3% of patients), while use of typical antipsychotics was slightly more frequent in the general wards (69.2% typical vs. 60.4% atypical; Table 1).

The antipsychotics prescribed and frequencies are shown in Table 2. Of the atypical antipsychotics, clotiapine and olanzapine were most popular. There were differences in choice of typical antipsychotics on the men's and women's wards. Zuclopenthixol was most common on the men's ward and haloperidol in the women's ward. Although typical antipsychotics were infrequently used on the geriatrics ward, zuclopenthixol and haloperidol were favored.

**Table 2:** Types of antipsychotics by ward.

	Drug/generic	Ward			
		Men N = 253	Women N = 230	Geriatric N = 121	Total N = 604
**Typical antipsychotics**	Chlorpromazine	0.4% (1)	1.7% (4)	0.0% (0)	0.8% (0)
	Flupentixol	0.0% (0)	0.0% (0)	0.8% (1)	0.2% (1)
	Fluphenazine	13.8% (35)	17.4% (40)	11.6% (14)	14.7% (89)
	Haloperidol	17.0% (43)	38.7% (89)	5.8% (7)	23.0% (139)
	Levomepromazine	1.2% (3)	0.9% (2)	0.0% (0)	0.8% (5)
	Penfluridol	0.0% (0)	1.3% (3)	0.8% (1)	0.7% (4)
	Perhphenazine	6.3% (16)	13.5% (31)	3.3% (4)	8.4% (51)
	Zuclopenthixol	28.5% (72)	17.8% (41)	7.4% (9)	20.2% (122)
**Atypical antipsychotics**	Amisulpride	6.7% (17)	2.6% (6)	10.7% (13)	6.0% (36)
	Aripiprazole	2.0% (5)	0.9% (2)	6.6% (8)	2.5% (15)
	Asenapine	0.8% (2)	0.9% (2)	0.8% (1)	0.8% (5)
	Clotiapine	16.6% (42)	23.9% (55)	6.6% (8)	17.4% (105)
	Clozapine	4.7% (12)	1.3% (3)	4.1% (5)	3.3% (20)
	Olanzapine	9.1% (23)	29.6% (68)	9.9% (12)	17.1% (103)
	Paliperidone	0.0% (0)	0.4% (1)	0.8% (1)	0.3% (2)
	Palperidone	3.6% (9)	0.4% (1)	19.8% (24)	5.6% (34)
	Quetiepine	7.9% (20)	8.3% (19)	5.8% (7)	7.6% (46)
	Risperidone	11.1% (28)	7.8% (18)	29.8% (36)	13.6% (82)
	Sertindole	0.4% (1)	0.0% (0)	0.0% (0)	0.2% (1)
	Ziprasidone	1.6% (4)	0.9% (2)	1.7% (2)	1.3% (8)

On average, patients received 2.4 types of drugs (median 2) other than valproate. Patients in the general male and female wards received significantly more types of drugs (2.5±0.9 vs. 2.3±0.8, respectively; p = 0.017) than the geriatric patients (1.4±0.6; p<0.001). There was no difference between men and women in the number of drug types received. Whereas 31.6% of patients (N = 191) were prescribed only typical antipsychotic medications, 36.4% (N = 220) were prescribed only atypical antipsychotic medications and 29.1% received both typical and atypical antipsychotic medications (APP). Of those patients with APP, zuclopenthixol (36.9%, N = 65), haloperidol (34.1%, N = 60) and fluphenazine (21.6%, N = 38) were the most commonly prescribed typical antipsychotics. Of those receiving APP, the most common atypical antipsychotics prescribed were clotiapine (40.9%, N = 72), quetiepine (13.6%, N = 24) and amisulpride (8.0%, N = 14). At least one mood stabilizer, including sodium valproate was prescribed for 157 (26%) of the individuals included in the study.

Overall, 111 patients (18.4%) received sodium valproate. Frequencies and percentages of sodium valproate use by ward and diagnosis are shown in Table 1. There were no gender differences within the men's and women's wards between patients who received sodium valproate and those who did not (χ^2^_(1)_ = 0.618, p>0.05). Sodium valproate use was significantly more common in patients in the general wards (20.1%) than among the geriatric patients (11.6%; p<0.05) and in patients with schizoaffective disorder (35.2%) vs. schizophrenia (14.1%; p<0.001) (Table 3).

**Table 3:** Frequency and percentage of sodium valproate use by ward and diagnosis.

Diagnosis	Ward		
	Men & Women % (N)	Geriatric % (N)	Total^a^ % (N)
**Schizophrenia**	16.1% (62) N = 385	6.2% (6) N = 97	14.1% (68) N = 482
**Schizoaffective**	35.7% (35) N = 98	33.3% (8) N = 24	35.2% (43) N = 122
**Total** b	20.1% (97) N = 483	11.6% (14) N = 121	18.4% (111) N = 604

a Significant dependency between ward and sodium valproate use: χ^2^ = 4.674, p = 0.031.

b Significant dependency between diagnosis and sodium valproate use: χ^2^_(1df)_ = 29.0, p<0.001.

Sodium valproate use was similar among patients who received antipsychotics, antidepressants and anxiolytics, ranging between 14% and 22.2% (Table 4). It was also more commonly prescribed for patients receiving APP. Sodium valproate was combined with another mood stabilizer in seven patients.

**Table 4:** Frequency and percentage of sodium valproate use by number and type of drugs.

Drug types	Prescribed sodium valproate % (N)
**Typical antipsychotics** N = 367	19.1% (70)
** Number**	
1 (N = 320)	18.8% (60)
2 (N = 45)	22.2% (10)
3 (N = 2)	0.0% (0)
**Atypical antipsychotics** N = 396	19.7% (78)
** Number**	
1 (N = 336)	19.0% (64)
2 (N = 59)	23.7% (14)
3 (N = 1)	0.0% (0)
**Antidepressants** N = 34	17.6% (6)
** Number**	
1 (N = 32)	18.8% (6)
2 (N = 2)	0.0% (0)
**Anxiolytics** N = 273	21.6% (59)
** Number**	
1 (N = 263)	22.1% (58)
2 (N = 10)	10.0% (1)
**Mood stabilizers** a N = 53	13.2% (7)
** Number**	
1 (N = 50)	14.0% (7)
2 (N = 3)	0.0% (0)
**Sleeping Pills** N = 345	17.4% (60)

a Other than sodium valproate.

In the logistic regressions, the odds ratios (OR) describing the relative risk of using sodium valproate with gender, diagnosis and ward were all greater than one (Tables 5 and 6). Diagnosis was associated with the highest risk estimate. Patients with a diagnosis of schizoaffective disorder were 3.7 times more likely to receive sodium valproate than those diagnosed with schizophrenia. Gender was not a significant predictor of sodium valproate use. The difference between the general and geriatric wards was not significant. The number of drug groups was not significant in predicting the use of sodium valproate (OR 1.14, 95% CI: 0.95–1.38; P = 0.151) (Table 5). However, in the logistic regression (Table 6) including gender, diagnosis, ward and APP, the use of APP was significant (OR = 1.63; 95% CI: 1.03–2.61; P = .04).

**Table 5:** Logistic regression predicting the use of sodium valproate by gender, ward, diagnosis and number of drugs.

Variable	OR	95% CI	P-value
Gender	1.26	(0.82–1.95)	0.298
Diagnosis	3.55	(2.22–5.65)	0.000
Ward	1.81	(0.97–3.39)	0.062
Number of drugs	1.14	(0.95–1.38)	0.151

**Table 6:** Logistic regression predicting the use of sodium valproate by gender, diagnosis, ward and number of antipsychotics.

Variable	OR	95% CI	P-value
Gender	1.45	(0.92–2.27)	0.108
Diagnosis	3.65	(2.28–5.84)	0.000
Ward	1.81	(0.97–3.37)	0.061
APP	1.63	(1.03–2.61)	0.039

## Discussion

This study assessed the extent of off-label use of sodium valproate and APP in several wards in a major psychiatric facility in Israel. The rates of mood stabilizers and sodium valproate use were very similar to those reported in Asia (24% for mood stabilizers and 16.7% for sodium valproate), but lower than those reported in the US, where approximately half of schizophrenic patients received mood stabilizers and about a third received sodium valproate [1], [9], [10], [14]. In the New York State hospital system, in 1996, approximately half of all patients diagnosed with bipolar or schizoaffective disorder and 28% of all patients diagnosed with schizophrenia, were prescribed valproate. For patients with schizophrenia, that number increased to 35% by 2001 [10], [11]. In a cross-sectional survey of in-patients 18 to 65 years of age from the United Kingdom, 28.5% were receiving an off-label mood stabilizer [17]. Of those patients, 28.2% had a diagnosis of schizophrenia and 17% had a diagnosis of schizoaffective disorder. Thus, off-label use of mood stabilizers was very common in this population of inpatients with severe mental illness. These differences from the current study, where 14% and 35% of patients with schizophrenia and schizoaffective disorder, respectively received sodium valproate, might be due to differences in patient population and case-mix. Alternatively, it might reflect variations in the organizational or cultural approaches of the treating physicians to the management of schizophrenia and to the legitimacy of off-label use in general or of sodium valproate in particular. Of particular importance, in the United States, off-label use is not regulated, leaving decisions in the hands of the treating physician. In Israel, however, physicians must obtain permission to use a drug outside its label indication. Although the procedure for hospitalized patients does not require individual authorization for each patient, but rather grants comprehensive approval for all patients who require the medication for the requested indication, the very existence of such a process might increase physicians' awareness of its off-label status and deter use.

Unsurprisingly, use of sodium valproate was considerably more prevalent in patients with schizoaffective disorder. Schizoaffective disorder presents as an intermediate form of psychotic disorder between schizophrenia and bipolar disorder, although unipolar versions of schizoaffective disorder have been described [18], [19]. Therefore, treatment for patients with this disorder revolves around a combination of antipsychotics, for the psychotic symptoms and either anti-depressants or mood stabilizers for the affective symptoms [20], [21]. Affective symptoms are not dominant in patients with schizophrenia and mood stabilizers are generally used only for controlling impulsivity or violence or as augmentation for patients who are unresponsive to the usual treatment. Sodium valproate is used for individuals with schizophrenia with violent episodes, because it is reported to decrease aggression [22]. It is used in treatment of resistant schizophrenia because there is a great need for adjuncts to antipsychotic drugs [9]. Several studies have reported some evidence of efficacy [22]–[26]. It is understood that GABA-ergic drugs such as valproate have a potential role in the treatment of schizophrenia, as they down-regulate dopamine [27]. Mesolimbic dopamine hyperactivity is considered one of the main reasons for the development of positive symptoms in schizophrenia. It should be noted that despite the lack of hard evidence of effectiveness for either indication, mood stabilizers are recommended for schizoaffective disorder, but not for schizophrenia. However, it can be difficult to differentiate the two disorders. Even the distinction between schizophrenia and bipolar disorder has been challenged based on findings of a partially shared genetic susceptibility pattern. In addition, psychotic symptoms are prevalent in mania and affective symptoms are common in patients with schizophrenia [28].

In this study, the use of sodium valproate was almost twice as common in patients in the general wards as among the geriatric patients. This is similar to other reports, where patients treated with mood stabilizers and/or sodium valproate were on average younger [1], [14]. It is possible that younger patients have more positive symptoms and an increased likelihood of aggressive or impulsive behavior, requiring the addition of mood stabilizers to their treatment. Alternatively, the relation between age and ward may not be direct. It is possible that the difference is not related to the age of the individual patients per se, but rather to different management of psychiatric disorders in geriatric wards. Given the physiological (and pathological) changes associated with aging, it is logical to avoid, if possible, polypharmacy and drugs with a narrow therapeutic range in this age group. Moreover, studies have found an increase in adverse events (falls, infection, gastrointestinal disorders) among patients treated with sodium valproate [29].

Previous studies have shown a relationship between indicators for disease severity or patient violence and the use of mood stabilizers. Sim et al. [14] reported a correlation with aggression and antipsychotic polytherapy and Pickar et al. [1] reported a correlation with a history of violence and higher Positive and Negative Syndrome Scale general psychopathology scores. This study did not find a correlation with the number of non-valproate drug types. The use of multiple types of drugs may not be a good stand-in for disease severity (i.e. used for augmentation), but rather reflects the different clinical symptoms a patient manifests. However, use of APP, the combined use of typical and atypical antipsychotics, was associated with valproate use. We found a prevalence rate of APP of 36.4%. This is higher than the prevalence reported in North America (16%), Oceania (16.4%), and Europe (23%), but comparable to that of Asia (32%) [30].

The use of APP is generally discouraged and guidelines suggest it should only be used as a last resort after clozapine (the standard of care for treatment of refractory psychotic illness) [31]–[36]. This is because, in addition to the lack of evidence base, the implications and consequences (side effects, dosage, interactions, etc.) are unknown. In this context, it is also worth noting that clozapine was infrequently prescribed (3.3%, N = 20). We suggest that use of APP is an indicator of refractory psychotic illness and therefore associated with valproate use. Alternatively, its use may reflect a predisposition by the prescribing doctor to use non-evidence-based treatments.

An interesting, although unrelated finding was the significant difference in the use of typical and atypical antipsychotics between general and geriatric patients. Atypical antipsychotics are more effective in treating negative symptoms, which are more commonly found in elderly patients with schizophrenia [37]. Typical and atypical antipsychotics also have vastly different safety profiles. The most significant side-effects of typical neuroleptics are neurological, while atypical neuroleptics are known to cause metabolic changes, including obesity, hyperlipidemia and diabetes. Because geriatric patients require lower doses of the atypical antipsychotics, the side-effects occur less frequently. For the elderly, the most frequently reported adverse side-effects were extrapyramidal symptoms. In most cases, the atypical antipsychotics had fewer extrapyramidal symptoms than did typical antipsychotics [38].

The preponderance of off-label use of sodium valproate for schizophrenia and schizoaffective disorder can have significant clinical and economic effects. Currently, there is no robust evidence regarding the effectiveness of sodium valproate in the standard treatment of these disorders [29]. Therefore, it is possible that, for some of those patients, adding it to their treatment regimen does not confer benefits, but rather exposes them to potential harm. While there is evidence that adding sodium valproate can benefit the most severe and unmanageable patients, it is unclear whether this reason alone can account for the rate of use found in this study.

Adding sodium valproate increases the costs for medications for schizophrenia, but to date there is no evidence that it reduces other expenditures, such as those related to hospitalizations [39]. Furthermore, in healthcare settings such as in Israel, where healthcare providers (Health Maintenance Organizations) are in part budgeted according to the expected utilization of medical technologies, off-label use adds a new, unplanned, and therefore unbudgeted population of users. While sodium valproate is an inexpensive drug, the prevalence of schizophrenia means that its addition can have a considerable impact on a provider's budget. This may also be true of APP. In addition to the paucity of clinical evidence supporting both practices, there is considerable concern and uncertainty regarding possible increases in side effects, higher than necessary (or approved) total dosage, drug-drug interactions, increased mortality and cost involved [30].

### Limitations

This study has several limitations. First, data was collected from three wards in one hospital, and therefore the findings might not be generalizable, especially to schizophrenic patients in the community. Secondly, the data available in the database system used is more administrative than clinical, which limits the ability to truly appreciate and refine the correlation between patients' clinical status and sodium valproate use. This includes a lack of medical history in order to understand the number of changes in medications before utilizing off-label options. In addition, diagnoses were based on the diagnosis entered by the treating physician in the patient record system according to ICD diagnostic criteria and not on diagnostic interviews. However, such broad determinations may be more useful for health policy and management purposes.

### Conclusions

Our data indicate that further study is necessary to determine the clinical considerations behind the use of sodium valproate in patients with psychotic disorders in Israel. Given the limited efficacy evidence available, more research is necessary to determine whether it is being prescribed appropriately for patients with schizoaffective disorder and for those with treatment-resistant schizophrenia.

## References

[1] Pickar D, Vinik J, Bartko J (2008) Pharmacotherapy of schizophrenic patients: preponderance of off-label drug use. PLoS One 3: e3150. 18781198 10.1371/journal.pone.0003150 PMC2522284

[2] Radley DC, Finkelstein SN, Stafford RS (2006) Off-label prescribing among office-based physicians. Arch Intern Med 166: 1021–1026. 16682577 10.1001/archinte.166.9.1021

[3] Healy D, Nutt D (1998) Prescriptions, licenses and evidence. Psychiatr Bull 22: 680–684.

[4] Wittich CM, Burkle CM, Lanier WL (2012) Ten common questions (and their answers) about off-label drug use. Mayo Clin Proc 87: 982–990. 22877654 10.1016/j.mayocp.2012.04.017 PMC3538391

[5] Leslie DL, Mohamed S, Rosenheck RA (2009) Off-Label use of antipsychotic medications in the department of veterans affairs health care system. Psychiatr Serv 60: 1175–1181. 19723731 10.1176/ps.2009.60.9.1175

[6] Chen DT, Wynia MK, Moloney RM, Alexander GC (2009) U.S. physician knowledge of the FDA-approved indications and evidence base for commonly prescribed drugs: results of a national survey. Pharmacoepidemiol Drug Safety 18: 1094–1110. 10.1002/pds.1825 19697444

[7] Conley RR, Kelly DL (2001) Management of treatment resistance in schizophrenia. Biol Psychiatry 50: 898–911. 11743944 10.1016/s0006-3223(01)01271-9

[8] Citrome L (2004) What role for mood stabilizers? Current Psychiatry 3: 23–39.

[9] Schwarz C, Volz A, Li C, Leucht S (2008) Valproate for schizophrenia. Cochrane Database of Systematic Reviews DOI:10.1002/14651858.CD004028.pub3. 10.1002/14651858.CD004028.pub3 18646098

[10] Citrome L, Levine J, Allingham B (2000) Changes in use of Valproate and other mood stabilizers for patients with schizophrenia from 1994 to 1998. Psychiatr Serv 51: 634–639. 10783182 10.1176/appi.ps.51.5.634

[11] Citrome L, Jaffe A, Levine J, Allingham B (2002) Use of mood stabilizers among patients with schizophrenia, 1994–2001. Psychiatr Serv 53: 1212. 12364663 10.1176/appi.ps.53.10.1212

[12] Wessels T, Grünler D, Bunk C, Specka M, Gastpar M, et al (2007) Changes in the treatment of acute psychosis in a German public hospital from 1998 to 2004. Psychiatr Q 78: 91–99. 17351755 10.1007/s11126-006-9030-y

[13] Davids E, Bunk C, Specka M, Gastpar M (2006) Psychotropic drug prescription in a psychiatric university hospital in Germany. Prog Neuropsychopharmacol Biol Psychiatry 30 (6): 1109–1116. 16737763 10.1016/j.pnpbp.2006.04.015

[14] Sim K, Yong KH, Chan YH, Tor PC, Xiang YT, et al (2011) Adjunctive mood stabilizer treatment for hospitalized schizophrenia patients: Asia psychotropic prescription study (2001–2008). Int J Neuropsychopharmacol 14: 1157–1164. 21557883 10.1017/S1461145711000563

[15] Citrome L (2009) Adjunctive lithium and anticonvulsants for the treatment of schizophrenia: what is the evidence? Expert Rev Neurother 9: 55–71. 19102669 10.1586/14737175.9.1.55

[16] European Medicines Agency. Valproate. Available: http://www.ema.europa.eu/ema/index.jsp?curl=pages/medicines/human/referrals/Valproate/human_referral_000187.jsp&mid=WC0b01ac0580024e9a&murl=menus/regulations/regulations.jsp&jsenabled=true. Accessed May 23, 2013.

[17] Haw C, Stubbs J (2005) A survey of the off-label use of mood stabilizers in a large psychiatric hospital. J Psychopharmacol 19: 402–407. 15982996 10.1177/0269881105053307

[18] Benabarre A, Vieta E, Colom F, Martinez-Aran A, et al (2001) Bipolar disorder, schizoaffective disorder and schizophrenia: epidemiologic, clinical and prognostic differences. Eur Psychiatry 16: 167–172. 11353595 10.1016/s0924-9338(01)00559-4

[19] Murru A, Pacchiarotti I, Nivoli AMA, Grande I, Colom F, et al (2011) What we know and what we don't know about the treatment of schizoaffective disorder. European Neuropsychopharmacology 21: 680–690. 21565468 10.1016/j.euroneuro.2011.03.001

[20] Levinson DF, Umapathy C, Musthaq M (1999) Treatment of schizoaffective disorder and schizophrenia with mood symptoms. Am J Psychiatry 156: 1138–1148. 10450252 10.1176/ajp.156.8.1138

[21] Olfson M, Marcus SC, Wan GJ (2009) Treatment patterns for schizoaffective disorder and schizophrenia among Medicaid patients. Psychiatr Serv 60: 210–216. 19176415 10.1176/ps.2009.60.2.210

[22] Citrome L, Casey DE, Daniel DG, Wozniak P, Kochan LD, et al (2004) Adjunctive divalproex and hostility among patients with schizophrenia receiving olanzapine or risperidone. Psychiatr Serv 55: 290–294. 15001730 10.1176/appi.ps.55.3.290

[23] Casey DE, Danie DG, Tamminga C, Kane JM, Tran-Johnson T, et al (2009) Divalproex ER combined with olanzapine or risperidone for treatment of acute exacerbations of schizophrenia. Neuropsychopharmacology 34: 1330–1338. 19052541 10.1038/npp.2008.209

[24] Meltzer HY, Bonaccorso S, Bobo WV, Chen Y, Jayathilake K (2011) A 12-month randomized, open-label study of the metabolic effects of olanzapine and risperidone in psychotic patients: influence of valproic acid augmentation. J Clin Psychiatry 72: 1602–1610. 21813074 10.4088/JCP.10m05997

[25] Suzuki T, Uchida H, Takeuchi H, Nakajima S, Nomura K, et al (2009) Augmentation of atypical antipsychotics with valproic acid. An open-label study for most difficult patients with schizophrenia. Hum Psychopharmacol 24: 628–638. 19946935 10.1002/hup.1073

[26] Kelly DL, Conley RR, Feldman S, Yu Y, McMahon RP, et al (2006) Adjunct divalproex or lithium to clozapine in treatment-resistant schizophrenia. Psychiatr Q 77: 81–95. 16397757 10.1007/s11126-006-7963-9

[27] Wassef AA, Dott SG, Harris A, Brown A, O'Boyle M, et al (2000) Randomized, placebo-controlled pilot study of divalproex sodium in the treatment of acute exacerbations of chronic schizophrenia. J Clin Psychopharmacol 20: 357–361. 10831024 10.1097/00004714-200006000-00011

[28] Qian K, Di Lieto A, Corander J, Auvinen P, Greco D (2012) Re-analysis of bipolar disorder and schizophrenia gene expression complements the Kraepelinian dichotomy. Adv Exp Med Biol 736: 563–577. 22161352 10.1007/978-1-4419-7210-1_33

[29] Lonergan E, Luxenberg J (2009) Valproate preparations for agitation in dementia. Cochrane Database Syst Rev (3): CD003945. 10.1002/14651858.CD003945.pub3 19588348

[30] Gallego JA, Bonetti J, Zhang JP, Kane JM, Correll CU (2012) Prevalence and correlates of antipsychotic polypharmacy from the 1970s to 2009: a systematic review and metaregression. Schizophr Res 138: 18–28. 22534420 10.1016/j.schres.2012.03.018 PMC3382997

[31] Buchanan RW, Kreyenbuhl J, Kelly DL, Noel JM, Boggs DL, et al (2010) Schizophrenia Patient Outcomes Research Team (PORT) The 2009 schizophrenia PORT psychopharmacological treatment recommendations and summary statements. Schizophr Bull 36: 71–93. 19955390 10.1093/schbul/sbp116 PMC2800144

[32] National Institute for Clinical Excellence (2003) Schizophrenia: full national guideline on core interventions in primary and secondary care. Leicester (UK) and London: The British Psychological Society and The Royal College of Psychiatrists; 2003.

[33] Moore TA, Buchanan RW, Buckley PF, Chiles JA, Conley RR, et al (2007) The Texas Medication Algorithm Project antipsychotic algorithm for schizophrenia: 2006 update. J Clin Psychiatry 68: 1751–1762. 18052569 10.4088/jcp.v68n1115

[34] McGorry PD (2005) Royal Australian and New Zealand College of Psychiatrists clinical practice guidelines for the treatment of schizophrenia and related disorders. Aust N Z J Psychiatry 39: 1–30. 15660702 10.1080/j.1440-1614.2005.01516.x

[35] Falkai P, Wobrock T, Lieberman J, Glenthoj B, Gataz WF, et al (2005) World Federation of Societies of Biological Psychiatry (WFSBP)–guidelines for biological treatment of schizophrenia, part 1: acute treatment of schizophrenia. World J Biol Psychiatry 6: 132–191. 16173147 10.1080/15622970510030090

[36] Lehman AF, Lieberman JA, Dixon LB, McGlashan TH, Miller AL, et al. (2004) Practice guideline for the treatment of patients with schizophrenia, second edition. Am J Psychiatry (Suppl 2): 1–56. 15000267

[37] Hartling L, Abou-Setta AM, Dursun S, Mousavi SS, Pasichnyk D, et al (2012) Antipsychotics in adults with schizophrenia: comparative effectiveness of first-generation versus second-generation medications: a systematic review and meta-analysis. Ann Intern Med 157: 498–511. 22893011 10.7326/0003-4819-157-7-201210020-00525

[38] Abou-Setta AM, Mousavi SS, Spooner C, Schouten JR, Pasichnyk D, et al. (2012) First-Generation Versus Second-Generation Antipsychotics in Adults: Comparative Effectiveness [Internet]. AHRQ Comparative Effectiveness Reviews. Report No. 12-EHC054-EF. Rockville (MD): Agency for Healthcare Research and Quality (US). 23035275

[39] Chen H, Kennedy WK, Dorfman JH, Fincham JE, Reeves J, et al (2007) The effect of adjunctive mood stabilizers on antipsychotic utilization pattern and health resource utilization for Medicaid enrollees with schizophrenia. Curr Med Res Opin 23: 1351–1365. 17559734 10.1185/030079907X187883

